# Two Novel Polysaccharides in *Psoralea corylifolia* L and anti-A549 Lung Cancer Cells Activity *In Vitro*

**DOI:** 10.3390/molecules24203733

**Published:** 2019-10-16

**Authors:** Zhenhua Yin, Wei Zhang, Juanjuan Zhang, Huili Liu, Qingfeng Guo, Lin Chen, Jinmei Wang, Wenyi Kang

**Affiliations:** 1 Henan Joint International Research Laboratory of Drug Discovery of Small Molecules, Zhengzhou 450063, China; yinzhenhua1000@126.com (Z.Y.); zhangjuan8908@163.com (J.Z.); huililiu@infm.hhstu.edu.cn (H.L.); guoqf.2008@163.com (Q.G.); lchenchina@163.com (L.C.); 2 Zhengzhou Key Laboratory of Medicinal Resources Research, Huanghe Science and Technology College, Zhengzhou 450063, China; zzzwwwqq@126.com; 3 National R & D Center for Edible Fungus Processing Technology, Henan University, Kaifeng 475004, China

**Keywords:** *Psoralea corylifolia* L, polysaccharides, structure, antitumor activity

## Abstract

Two novel water soluble heteroglycan (PCp-I and PCp-II) with anti-A549 lung cancer cells activity were isolated from *Psoralea corylifolia* L. Their average molecular weights were 2.721 × 10^4^ and 2.850 × 10^4^. PCp-I and PCp-II had the same monosaccharide composition, but their molar ratios were different. Based on methylation and NMR spectroscopy, the part structure of PCp-I was identified. The results of scanning electron microscope (SEM) showed that PCp-I had an irregular porous structure and PCp-II was flaky and irregularly curved. The results of thermogravimetry-differential scanning calorimetry (TG-DSC) showed that PCp-I and PCp-II had good thermal stability. Furthermore, PCp-I and PCp-II exhibited significant anti-A549 lung cancer cells activity (IC_50_ = 64.84 and 126.30 μM) *in vitro*.

## 1. Introduction

Polysaccharides, as an important carbohydrate in nature, are from different sources, such as plant, fungi, and algae, and they exhibit different chemical and biological activities, depending on the structure and nature of the monosaccharides [1]. Due to the physicochemical and functional properties of polysaccharides, i.e., their water retention ability, filming capacity, antioxidant, anti-microbial, immunomodulatory, anti-cancer, constipation, and antithrombus activities [2,3,4,5,6,7], polysaccharides are used in a wide variety of industrial applications, such as food, pharmaceuticals, and textiles. However, inrecent years, due to its high molecular weight, polysaccharides have poor water solubility, so there has been increasing interest in water-soluble polysaccharides as an important class of bioactive substances that may compete with traditional polysaccharides due to their potential biological activities and processing properties. 

*Psoralea corylifolia* L. (PC), belonging to Leguminosae, is one of the most popular traditional Chinese medicines used for psoriasis and vitiligo [8,9]. Phytochemical researches show that *P. corylifolia* contains coumarins, flavonoids, and monoterpene phenols [10,11]. As for the polysaccharides of *P. corylifolia*, the studies on polysaccharides from *P. corylifolia* purification and their structural characteristics and biological activities for *P. corylifolia* polysaccharides were few, and only a few scholars have conducted preliminary studies, for example Zheng C X et al. [12] initially studied the feasibility and the mechanism of the polysaccharide from *P. corylifolia* for the repair of articular cartilage defects in rabbits. Yang G and Li F S et al. [13,14] initially studied the immunomodulatory activities on normol mice of *P. corylifolia* fructus. crude polysaccharide, but the potential active components have not been identified. Recent studies suggested that extracts from *P. corylifolia* could inhibit the growth of ehrlich ascites tumor and lung cancer cells [15,16]. However, there is relatively little information pertaining to the purification and their structural characteristics and biological activities of water-soluble polysaccharides that were isolated from *P. corylifolia.*

At present, some scholars have found that the structure of polysaccharides is closely related to its biological activity [17,18]. Clarifying the structures of polysaccharides is an interesting goal to understand structure-activity relationships and causes of these biological activities. Therefore, in this study, polysaccharides of *P. corylifolia* were isolated by DEAE-52 cellulose column chromatography and Sephadex G-100 chromatography. The structures were identified by the methods of fourier transform-infrared spectroscopy (FT-IR), nuclear magnetic resonance (NMR), gas chromatography–mass (GC-MS), and scanning electron microscope (SEM). Furthermore, the antitumor activity towards anti-A549 lung cancer cells was assayed, aiming to provide fundamental information on the structure characterization and reveal the anti-A549 lung cancer cells activity, which provided theoretical basis for further research.

## 2. Results and Discussion

### 2.1. Isolation and Purification of Polysaccharides

**Figure 1:**
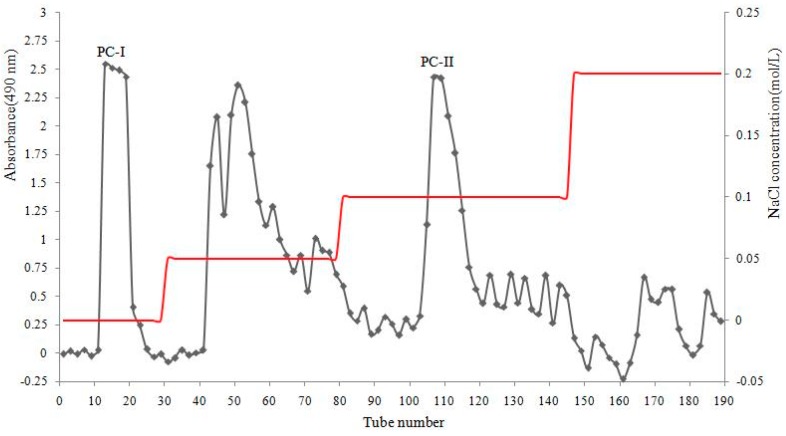
DEAE-52 chromatography of crude polysaccharide from *P. corylifolia.*

**Figure 2:**
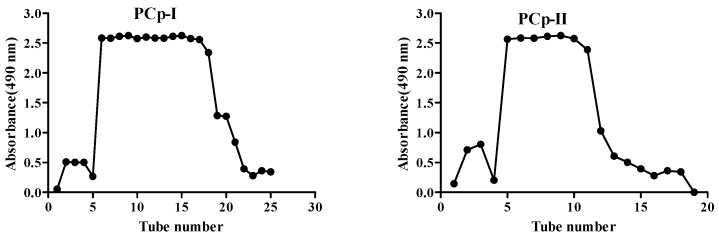
Sephadex G-100 chromatography of PC-I and PC-II.

The refined polysaccharide (80 g, yield of 1.82%) was fractionated by DEAE-52 cellulose column chromatography, eluted stepwise with ultra-pure water and different concentrations of aqueous sodium chloride, respectively, and ultra-pure water eluate was pooled as PC-I (yield of 18.04%), 0.1 mol/L NaCl eluate was pooled as PC-II (yield of 5.32%). Figure 1 showed the procedure. They were further purified by gel chromatography on a Sephadex G-100 column and PCp-I and PCp-II were obtained with yields of 39.71% and 36.80%, respectively (Figure 2). PCp-I was neutral polysaccharides that were eluted with water. PCp-II was acidic polysaccharides eluted with 0.1 mol/L NaCl through anion-exchange chromatography [19]. 

### 2.2. General Physicochemical Properties

Table 1 showed the general physicochemical properties of PCp-I and PCp-II. The total sugar contents of PCp-I and PCp-II were 96.35 ± 0.42 % and 97.07 ± 0.34 %, respectively, the protein content were 0.082 ± 0.002% and 0.077 ± 0.001%, respectively, which showed that PCp-I and PCp-II had high purity and little protein. PCp-I and PCp-II were white powder, soluble in water, and insoluble in ethanol, *n*-butanol, acetone, chloroform, and petroleum ether. Fehling reagent reaction and ferric chloride reaction were negative, which indicated that the two polysaccharides did not contain free monosaccharide and polyphenols, and the reaction of the two polysaccharides with I-KI reaction was also negative, indicating that the two polysaccharides were not starch polysaccharides.

### 2.3. Molecular Weight and Monosaccharide Composition Analysis

**Figure 3:**
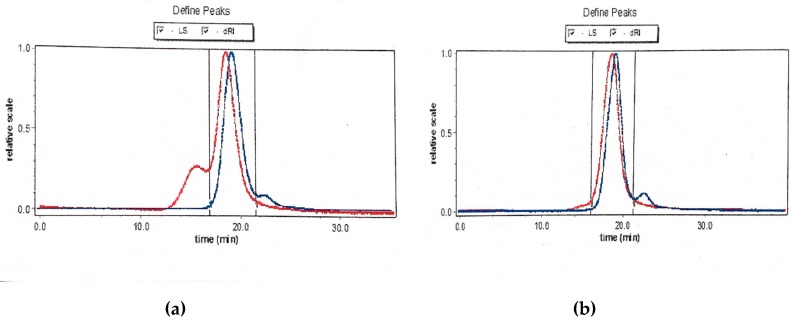
performance size-exclusion chromatography (HPSEC) elution profiles of PCp-I (**a**) and PCp-II (**b**).

The homogeneity and molecular weight of PCp-I and PCp-II were determined using high performance size-exclusion chromatography (HPSEC), and only a single peak was observed (Figure 3 and Table 2). The molecular weight was estimated to be approximately 2.721 × 10^4^ and 2.850 × 10^4^ g/mol, according to a standard calibration curve that was obtained from definite molecular weight dextrans. The Mn was 1.911 × 10^4^ and 2.339 × 10^4^ g/mol, respectively. The dispersion coefficient (Mw/Mn) was used to be a judgment as to whether the molecular weight was distributed uniformly or not. 

Gas Chromatography (GC) [20] and High-Performance Liquid Chromatography (HPLC) [21] were more commonly used to determine the monosaccharide composition. GC was the method for neutral sugar analysis with superb resolution and high sensitivity, however it required derivatization prior to analysis and it could not directly detect acid sugar [22]. Usually, GC analysis could give the accurate content of sugars in the polysaccharides. In our study, PCp-I and PCp-II were first hydrolyzed and acetylated, and then the monosaccharide compositions were measured by GC and identified by comparing the retention time of standards. In Table 3, PCp-I had a high amount of galactose and arabinose, and PCp-II mainly consisted of rhamnose, xylose, and galactose.

### 2.4. FT-IR Spectra Analysis

**Figure 4:**
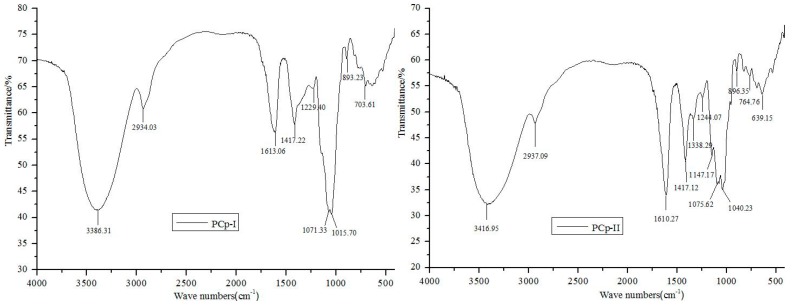
Fourier transform-infrared spectroscopy (FT-IR) spectra of PCp-I and PCp-II.

FT-IR spectroscopy was used to examine the main functional groups of carbohydrates [23]. Figure 4 showed the FT-IR spectra of PCp-I and PCp-II. In the FT-IR spectrum, a strong and broad band at 3386 and 3416 cm^−1^ were the characteristic of O–H stretching frequency, which was indicative of the strong inter- and intra-molecular interactions of the polysaccharide chains [24]. The peaks at 2934 and 2937 cm^−1^ were assigned to aliphatic C–H stretching [25]. The absorption peaks around 1610, 1417, and 1070 cm^−1^ were assigned to bending vibration of C–OH and C–O stretching of ether and anti-symmetric stretching band of C–O–C groups, respectively [26]. The *α* and *β* conformations of the carbohydrate could be determined by the peak position of the terminal carbon in the 950–750 cm^−1^ region, where 870–840 cm^−1^ correspond to *α* configuration and *β* configuration belong to 890 cm^−1^. The peaks that were observed at 896 and 893 cm^−1^ indicated that the PCp-I and PCp-II had a β-glycoside link [27].

### 2.5. Methylation Analysis

The linkage patterns of PCp-I and the corresponding percentages of alditol acetates were investigated while using methylation and GC-MS method. A major peak was observed in the GC profile (Data not shown), which was identified as 2,3,6-Tri-O-Me-Gal (35.66%), indicating that the main sugar residue of 1,4-linked-Gal*p* was present in PCp-I. The total percentage of terminal sugar residues (2.34% of T-Glc*p* and 6.74% of T-Gal*p*) was 9.08%. The sugar residues of 1,3-linked-Gal*p* (3.80%), 1,5-linked-Ara*f* (7.91%), 1,3,6-linked-Man*p* (3.09) and 1,4-linked-Man*p* (2.18%) were also detected (Table 4). In addition, the content of rhamnose was reduced and the xylose was not detected. These results indicated that the main sugar residues of PCp-I were galactose and arabinose. The ratio change might be due to the degradation of polysaccharide chain during the reduction procedure; however, the intrinsic reason still needs to be further discussed [22]. As a whole, the results of methylation were basically consistent with the monosaccharide composition analysis.

### 2.6. The Chemical Shifts Assignments of Different Linkage Patterns of PCp-I by NMR 

**Figure 5:**
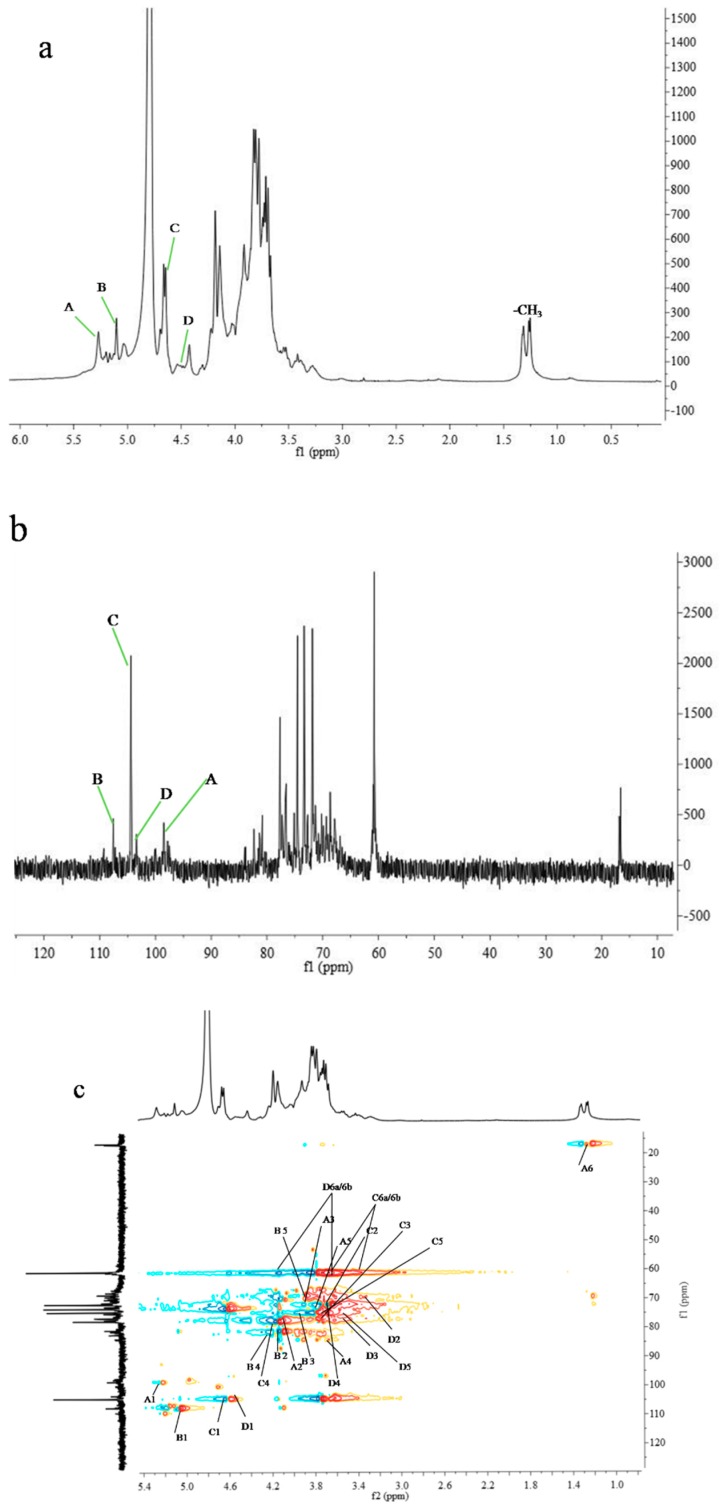

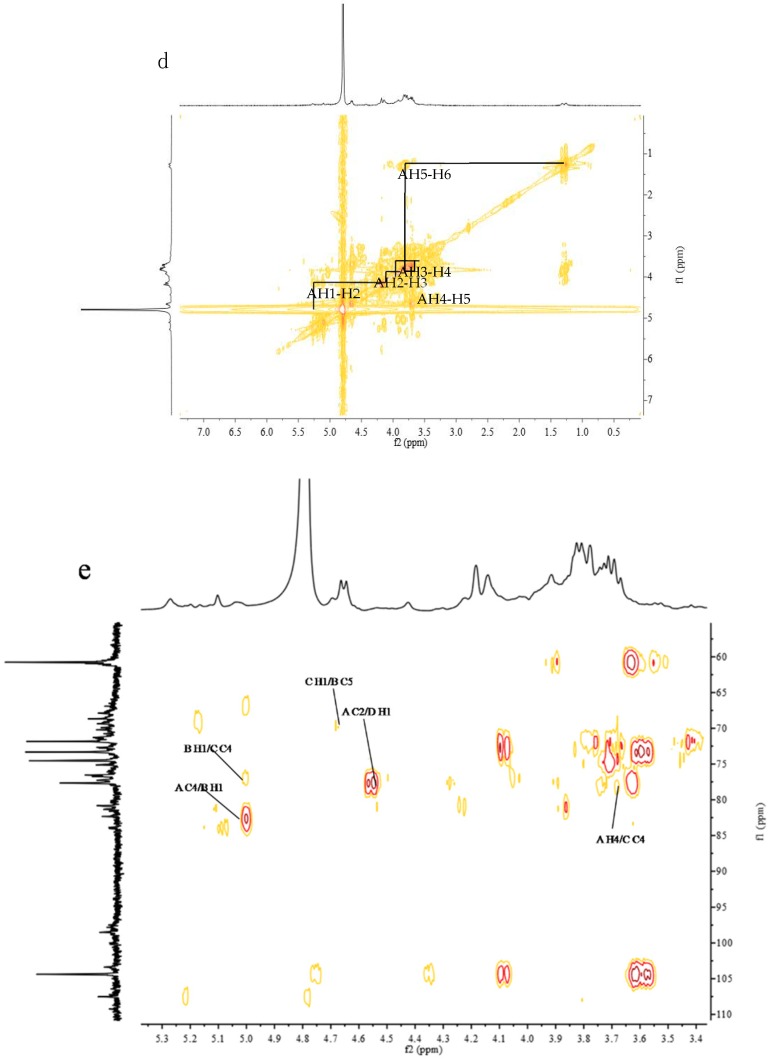
(**a**) ^1^H-NMR spectrum (400 MHz, D_2_O, 30 °C); (**b**) ^13^C NMR spectrum (100 MHz,.D_2_O, 30 °C); (**c**) ^1^H/^13^C HSQC correlation spectrum; (**d**) ^1^H/^1^H COSY correlation spectrum of PCp-1; and, (**e**) ^1^H/^13^C HMBC correlation spectrum.

NMR spectroscopy, including one-dimensional (1D) and two-dimensional (2D) NMR spectra, was conducted for the elucidation of the structural features of PCp-I. The ^1^H-NMR spectrum (Figure 5a) showed four signals in the anomeric region at *δ* 5.27(A), 5.10(B), 4.65(C), and 4.54 (D). They were designated as A to D residues, according to their decreasing proton chemical shift values. In the ^13^C-NMR spectrum (Figure 5b) four anomeric signals appeared at *δ*107.5, 104.4, 103.4, and 98.5. The other carbon signals were in the region *δ* 83.7-60.0. The anomeric carbon chemical shift values of residues A to D were correlated to the anomeric proton signals of residues from the HSQC spectrum (Figure 5c).The anomeric carbon signal at *δ*107.5 correlated to anomeric proton signal of B (*δ* 5.10), *δ*104.4 correlated to the signals C (*δ* 4.65), at 103.4 correlated to D (*δ* 4.54), at *δ* 99.9 correlated to A (*δ* 5.27) residues, respectively. All of the ^1^H and ^13^C signals (Table 5) were assigned by ^1^H-^13^C HSQC (Figure 5c) and ^1^H-^1^H COSY (Figure 5d) experiments. The signals around at (1.3 and 17 ppm) were assigned to the protons of the methyl group. The peaks in the HSQC (1.26, 17.17 ppm) (Figure 5c) indicated that PCp-I contained rhamnose residue.

Residue A: In the ^1^H spectrum, Residue A had an anomeric proton chemical shift at *δ* 5.27 ppm, with the low field anomeric signal showing that it was an α-linked residue with relatively low content in PCp-I. It was identified as→2,4)-α-Rha*p*-(1→. The complete ^1^H assignments were achieved through the COSY spectrum (Figure 5d), which were *δ* 4.13, 3.92, 3.66, 3.80, and 1.26 ppm for H-2, H-3, H-4, H-5, and H-6a/H6b, respectively (Table 5). The chemical shifts from C-1 to C-5 could be assigned from the ^1^H-^13^C HSQC spectrum, as shown in Table 5. All of the ^1^H and ^13^C chemical shifts that were assigned in this study (Table 5) were consistent with previous data [28], which corroborate the above assignments.

Residue B: Residue B had an anomeric proton signal at *δ*5.04 ppm. The proton resonances (Table 5a) of residue B from H-1 to H-6 had been assigned to the α-arabinofuranose residue [29]. The ^13^C signal for the anomeric carbon was observed at *δ*107.4 ppm. The carbon signals from C-2 to C-5 were identified from the HSQC spectrum as shown in Figure 5c and Table 5. The chemical shift of C-5 was decreased, which indicated that it was the value of methyl glycosides and residue B was (1,5)-α-Araf [30,31]. All of the ^1^H and ^13^C chemical shifts assigned in this study (Table 5a). Residue B was assigned as→5)-α-Ara*f*-(1→ by comparing with previous data [32] and the methylation analysis results. 

Residue C: The signals of residue E at *δ* 4.65 ppm and 104.4 ppm corresponded to an β-linked residue with high content in PCp-I. This residue was tentatively assigned as *β*-1,4-linked-Galp by comparing with the reported data and peak intensity [33]. The proton and corresponding ^13^C assignments of residue C were obtained from COSY and HSQC spectrums, as shown in Figure 5c,d and Table 5. All of the ^1^H and ^13^C chemical shifts of residue C were inconsistent with the previous reports [28], and the corresponding intensity was supported by the methylation analysis results.

Residues D: Residue D was analyzed with the same way. The proton and carbon shifts of residue E were fully identified according to ^1^H-^1^H COSY and HSQC spectras (Table 5). The results were compared with previous data and methylation analysis, residue D was assigned as β-Glc*p*-(1→ [26,34].

### 2.7. Sequence Analysis of Polysaccharide Chain by HMBC Spectrum

**Figure 6:**
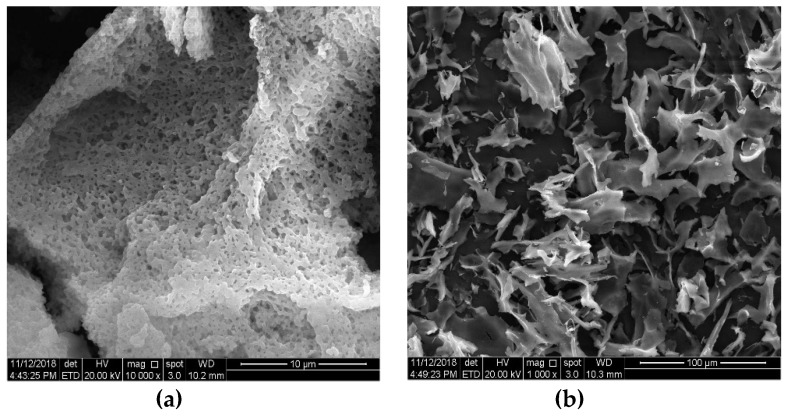
Photomicrographs of PCp-I (**a**) and PCp-II (**b**) as recorded by SEM.

Once the ^1^H and ^13^C chemical shifts of sugar residues were completely assigned, the sequences of these residues were determined by observing residual connectivities in HMBC spectrum (Figure 6e and Table 6). Cross peaks were found between H-4 of residue A (*δ* 3.66 ppm) and C-4 of residue C (*δ* 77.8 ppm), C-2 of residue A (δ 77.7 ppm) and H-1 of residue D (*δ* 4.54 ppm), and C-4 of residue A (*δ* 84.2 ppm) and H-1 of residue B (*δ* 5.04 ppm). The same way was used as those for residues of B, C, and D. Based on the monosaccharide composition, methylation and NMR spectroscopy, part structure of PCp-I was proposed. About some monosaccharide and methylation information were limited, so we cannot be inferred their location.

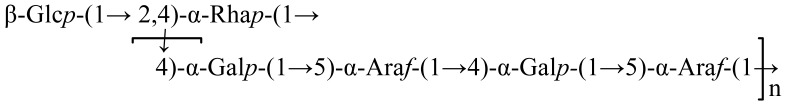


### 2.8. Molecular Morphology

SEM imaged the two polysaccharides to better understand the molecular morphology of PCp-I and PCp-II. PCp-I had an irregular porous structure, as shown in Figure 6. The dense network structure was presented as a whole. PCp-II was flaky and irregularly curved, and the surface was smooth, there were very small gaps between the crystals, so that the polysaccharides were not completely assembled, which indicated that there were repulsive forces between the molecules of the polysaccharides [35]. 

### 2.9. Thermal Stability Analysis

**Figure 7:**
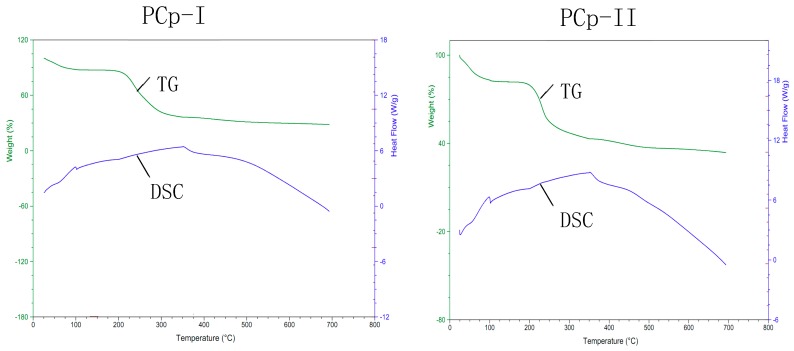
gravimetric and differential scanning calorimetric analysis of PCp-I and PCp-II.

Thermogravimetry (TG) and differential scanning calorimetry (DSC) measurements were used for studying the mass loss and thermal transitions in the course of heating under an inert atmosphere. Figure 7 and Table 7 illustrated the results. The TG experiments (Figure 7) showed two mass loss events for PCp-I and PCp-II, being the first near 100 °C, which might be attributed to the loss of adsorbed and structural water of both polysaccharides, as related by other authors [36]. The DSC experiments showed, for both polysaccharides, an endothermic event near 100 °C, absorbed heat were 98.10 and 236.80 J/g, probably due to the water evaporation, in agreement with TG analysis. The second mass loss event, with an onset temperature of 150.15 and 132.98 °C and a peak temperature of 419.44 and 386.80 °C for PCp-I and PCp-II, respectively, resulted in a weight loss of 51.97% and 39.21%, which might be attributed to the polysaccharide decomposition. The DSC experiments had good correlation with TG peak temperatures. The third mass loss event was a slow mass loss process, the decomposition process of both polysaccharides was basically over, and the final residual mass was 28.45% and 33.72%, respectively. The DSC experiments showed that the thermal decomposition temperatures of PCp-I and PCp-II were 355.23 °C and 359.36 °C, which was significantly higher than that of other polysaccharides [37,38], and showed that PCp-I and PCp-II had good thermal stability. 

### 2.10. PCp-I and PCp-II Inhibited the Proliferation of A549 Lung CancerCells

**Figure 8:**
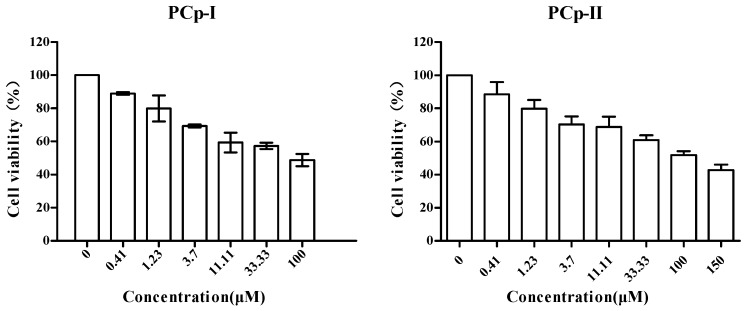
of PCp-I and PCp-II on cell viability of A549 lung cancer cells.

In the course of tumor progression, cancer cells undergo a number of characteristic changes, including the growth-inhibitory signals or ability of proliferation independently of exogenous growth-promoting [39]. Significant tumor inhibition on A549 lung cancer cells was observed at different concentrations of PCp-I and PCp-II as compared with the control group (Figure 8). PCp-I and PCp-II could decrease A549 cell viability in a dose-dependent manner. At the concentration of 100 μM, the cell viabilities were 48.77% and 51.87%, respectively. Comprehensive analysis, PCp-I and PCp-II had an inhibitory effect on A549 lung cancer cells (IC_50_ = 64.84 and 126.30 μM), but their activities were lower than that of cisplatin as positive control (IC_50_ = 11.00 μM). Han et al [5] found that a water-soluble pectic polysaccharide HCA4S1 that isolated from *Houttuynia cordata* might inhibit the proliferation of A549 lung cancer cell by inducing cell cycle arrest and apoptosis, and the expression of cleaved caspase 3 and cyclinB1 was observed to be upregulated after the treatment with this polysaccharide. It was further found that PCp-I had structural fragments that were similar to that of HCA4S1. At present, Lentinan and Ginseng polysaccharide had anti-tumor activity and been used in clinical [40,41]. These suggest that *P. corylifolia* polysaccharides are of potential value in the treatment of lung cancer. 

## 3. Materials and Methods

### 3.1. Materials and Chemicals

*Psoralea corylifolia* Linn. (PC) was purchased from Bozhou Yonggang Pieces Factory Co., Ltd. (Bozhou, China) which was identified by Changqin Li, the professor of pharmaceutical college of Henan University. The voucher specimens were deposited in the herbarium of Huanghe Science and Technology College. 

The monosaccharides standard substances (l(+)-rhamnose, l(+)-arabinose, d(+)-xylose, d(+)-mannose, d(+)-glucose, d-galactose) were purchased from the Dr Ehrenstorfer GmbH (Germany). DEAE-52 Cellulose was purchased from Saipuruisi Technology Co., LTD. (Beijing, China). Sephadex G-100 was purchased from GE Healthcare Bio-Science AB (Stockholm, Sweden). 

### 3.2. Preparation of Polysaccharides Sample

**Figure 9:**
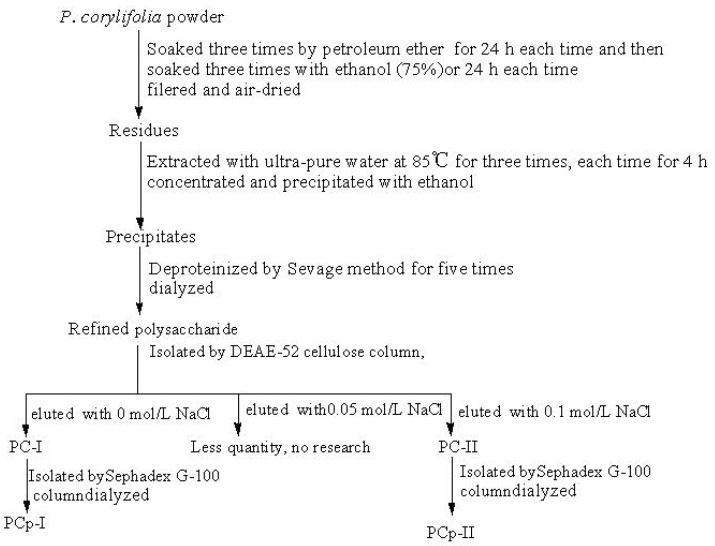
Isolation procedures of polysaccharide from *P. corylifolia*.

The extraction of polysaccharide from *P. corylifolia* (PC) was carried out according to our previous method [42], as demonstrated in Figure 9. The dried powder was successively soaked three times for 24 h each time with petroleum ether and 75% ethanol to eliminate some fat-soluble substances [43]. The soaked residue was extracted three times with ultra-pure water at 85 °C. Three volumes of ethanol was added to the water extraction solution to precipitate the crude polysaccharide. The Sevage method [44] was used to remove proteins. The DEAE-52 cellulose column (60 cm × 2.5 cm) and Sephadex G-100 column (100 cm × Φ 1.5 cm) chromatography were used to refine polysaccharide. 

### 3.3. General Physical and Chemical Properties

The total sugar and protein contents of polysaccharides were determined by the phenol- sulfuric acid [45] and coomassie brilliant blue G-250 [46] methods, respectively. The solubilities of polysaccharides in hot water, cold water, ethanol, *n*-butanol, acetone, chloroform, and petroleum ether were determined, feilin reagent reaction, ferric trichloride reaction, and iodine-potassium iodide reaction were also carried out [47].

### 3.4. Molecular Weight Determination 

The molecular weights of PCp-I and PCp-II were determined by HPSEC) according to Chinese Pharmacopoeia (2015 Edition, General Rules 0514) [48] in Beijing center for physical and chemical analysis.

### 3.5. Monosaccharide Composition Analysis

First, the derivatives (hydrolysis and acetylation) of PCp-I and PCp-II were carried out, and monosaccharide compositions were then analyzed by Gas Chromatography (GC, GC-2010, Shimadzu, Japan) equipped with a HP capillary column (30 m × 0.35 mm × 0.25 μm, Aglient Technologies, Inc., PaloAlto, Santa Clara, CA, USA) and a FID detector using the published method [49]. The standard monosaccharides (glucose, xylose, arabinose, rhamnose, mannose, ribose, fructose, and galactose) were derivatized and subjected to GC analysis in the same way. Monosaccharide composition of polysaccharides was identified by comparison with the chromatograms of standard monosaccharides, and the relative molar ratios were calculated by the method of area normalization.

### 3.6. Methylation Analysis 

Methylation analysis procedure was conducted according to the method of Ciucanu and Kerek [50] with slight modification [51]. The dried methylated sample was submitted to hydrolysis with trifluoroacetic acid (TFA), reduction with sodium borohydride (NaBH_4_), and acetylation with acetic anhydride to derive partially methylated alditol acetates (PMAA), which were analyzed by capillary GC-MS (Agilent Technologies, USA). A capillary column (30.0 × 250.0 μm × 0.25 μm) of DB-5 ms, held at 110 °C during injection for 1 min., then programmed at 5 °C/min. to 180 °C and held at this temperature for 4 min., at 3 °C/min. to 210 °C and held for 8 min. at 5 °C/min. to 230 °C and held for 3 min. was used for separation., 

### 3.7. FT-IR Spectral Analysis

The dried polysaccharides (PCp-I and PCp-II) were ground with spectroscopic grade KBr powder and then pressed into pellets for the analysis. The FT-IR spectrum was recorded in the range of 4000–400 cm^−1^ on a Thermo Scientific Nicolet iS5 FT-IR spectrophotometer (USA). 

### 3.8. Nuclear Magnetic Resonance (NMR) Spectroscopy Analysis

Polysaccharides (PCp-I and PCp-II) were exchanged with deuterium by lyophilizing against deuterium oxide (D_2_O) twice, and finally dissolved in D_2_O at room temperature for 4 h before NMR analysis. ^1^H and ^13^C-NMR spectra were recorded at 400 and 100 MHz, respectively, on a Bruker Avanced III 400 MHz NMR spectrometer (Karlsruhe, Germany) at 298 K. The homonuclear ^1^H-^1^H correlation (^1^H-^1^H COSY), heteronuclear multi-quantum relationship (HMQC), and heteronuclear multiple-bond correlation (HMBC) experiments were conducted by the standard Bruker pulse sequence.

### 3.9. Thermal Stability Analysis

TGA were obtained from a SDT Q600 instrument (TA Company, Milford, MA, USA), in the temperature range of 30–700 °C at a heating rate of 5 °C/min. under the nitrogen atmosphere. 

### 3.10. Molecular Morphology Observation

SEM (FEI Quanta 250 FEG, Hillsboro, OR, USA) was employed to observe the morphologies of the PCp-I and PCp-II samples that freeze-dried in the same way. The samples were coated with a thin gold film. The SEM images were observed at a voltage of 20.00 kV under the high vacuum condition.

### 3.11. Cell Culture and MTT Assay

The A549 cells were cultured in RPMI 1640 medium and incubated at 37 °C with 5% CO_2_ under a humidified atmosphere. A549 cells (2 × 10^4^ cells) were seeded in 96-well tissue culture plates and then cultured with PCp-I and II at different concentrations with three repeats for each concentration. After 48 h, 10 μL MTT was added into each well and maintained for 4 h at 37 °C. The insoluble violet formazan product was solubilized by the addition of 150 μL of DMSO. The absorbance was recorded at 490 nm by the Envision multimarker microporous plate detection system. Cisplatin was positive control. 

## 4. Conclusions

Two novel polysaccharides (PCp-I and PCp-II) were isolated and identified in *P. corylifolia* L. PCp-I and PCp-II were composed of rhamnose, arabinose, xylose, mannose, glucose, and galactose with different molar ratio, average molecular weight of were 2.721 × 10^4^ and 2.850 × 10^4^, respectively. PCp-I and PCp-II had good thermal stability and different microstructure. Further, PCp-I and PCp-II could inhibit A549 lung cancer cells activity (IC_50_ = 64.84 and 126.30 μM) in vitro. Based on methylation and NMR spectroscopy, part structure of PCp-I was proposed, as follows:

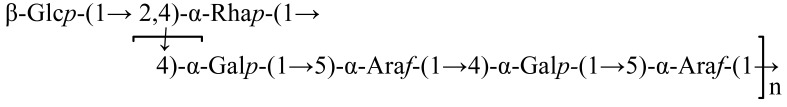


## Figures and Tables

**Table 1:** The general physicochemical properties of PCp-I and PCp-II.

Physicochemical Properties	PCp-I	PCp-II
Appearance	Straw yellow and fluffy	Straw yellow and fluffy
Solubility		
Hot water	Soluble	Soluble
Cold water	Soluble	Soluble
Ethanol,	Insoluble	Insoluble
*n*-Butanol	Insoluble	Insoluble
Acetone	Insoluble	Insoluble
Chloroform	Insoluble	Insoluble
Petroleum ether	Insoluble	Insoluble
Chemical Reaction		
Coomassie brilliant blue staining	+	+
Fehling reagent	-	-
Ferric chloride	-	-
I-KI	-	-

**Table 2:** Molecular weight of PCp-1 and PCp-4.

Samples	Molecular Weight (g/mol)		Mw/Mn
	Mw	Mn	
PCp-I	2.721 × 10^4^	1.911× 10^4^	1.424
PCp-II	2.850 × 10^4^	2.339× 10^4^	1.219

**Table 3:** Monosaccharide compositions of PCp-Iand PCp-II.

Polysaccharide	Major Monosaccharide Composition (Molar Ratio)					
	Rhamnose	Arabinose	Xylose	Mannose	Glucose	Galactose
PCp-I	1.65	4.47	2	2.06	0.946	24.76
PCp-II	2.79	1.97	7.52	0.283	0.187	6.62

**Table 4:** Results of the main methylation analysis of PCp-I.

Partially *O*-Methylalditol Acetate	SF(%)	Linkage Type
3-*O*-Me-Rha	0.19	→ 2,4)-Rha*p*-(1→
2,3-di-*O*-Me-Ara	7.91	→5-Ara*f*-(1→
2,4,6-Tri-*O*-Me-Gal	3.80	→3)-Gal*p*-(1→
2,3,4,6- tetra-*O*-Me-Gal	6.74	Gal*p*-(1→
2,3,6-Tri-*O*-Me-Gal	35.66	→4)-Gal*p*-(1→
2,3,4,6-tetra-*O*-Me-Glc	2.34	Glc*p*-(1→
2,4-di-*O*-Me-Man	3.09	→3,6)-Man*p*-(1→
2,3,6-di-*O*-Me-Man	2.18	→4)-Man*p*-(1→

Note: SF: % of peak area of *O*-methyl alditol acetates relative to total area, determined by GC–MS. Sorting is not related to peak-out time.

**Table 5:** The ^1^H-NMR and^13^C-NMR chemical shifts of PCp-I.

Glucosyl Residue	H-1/	H-2/	H-3/	H-4/	H-5/	H-6(a,b)/
	C-1	C-2	C-3	C-4	C-5	C-6
A	5.27	4.13	3.92	3.66	3.80	1.26
→ 2,4)-α-Rha*p*-(1→	98.5	77.7	71.9	84.2	72.1	17.2
B	5.04	4.15	3.96	4.23	3.88/3.92	-
→5)-α-Ara*f*-(1→	107.5	80.9	74.6	81.3	68.9	-
C	4.65	3.68	3.78	4.18	3.79	3.72/3.52
→ 4)-α-Gal*p*-(1→	104.4	71.9	76.6	77.8	74.6	60.9
D	4.54	3.31	3.56	3.71	3.54	3.68/3.95
β-Glc*p*-(1→	103.4	69.7	76.0	71.8	75.8	60.8

**Table 6:** The significant connectivities observed for the anomeric protons/carbons of PCp-1 in HMBC spectrum.

Sugar Residue	H-1/C-1(ppm)	Connectivities		
	δH/δC	δH/δC	Residue	Atom
A	3.66	77.8	C	C-4
	77.7	4.54	D	H-1
	84.2	5.04	B	H-1
B	5.04	77.8	C	C-4
C	4.65	68.9	B	C-5
	77.8	3.88	B	H-5

**Table 7:** The thermal gravimetric and differential scanning calorimetric analysis results of PCp-I and PCp-II.

DSC-TG Analytical Parameters		PCp-I	PCp-II
Phase I	Began-end temperature (°C)	24.74~150.15	24.74~132.98
	Enthalpy peak temperature (°C)	100.55 (98.10 J/g)	100.94 (263.8 J/g)
	Quality change (%)	10.34	15.05
Phase II	Began-end temperature (°C)	150.15~419.44	132.98~386.80
	Enthalpy peak temperature (°C)	355.23	359.36
	Quality change (%)	51.97	39.21
Phase III	Began-end temperature (°C)	419.44~693.75	386.80~693.75
	Quality change (%)	8.75	8.18
693.75	Residual quality (°C)	28.45	33.72
